# Caries risk in toddlers: assessing oral health, microbial factors, and maternal transmission

**DOI:** 10.3389/fdmed.2026.1847228

**Published:** 2026-06-22

**Authors:** Emma Scudero, Nolan Manning, Vanessa T. Mariscal, Molly McNulty, Pat Fidopiastis, Navid Fardanesh, Casey Heaney, Suzanne Phelan

**Affiliations:** 1 Center for Health Research, California Polytechnic State University, San Luis Obispo, CA, United States; 2 Community Health Centers of the Central Coast, San Luis Obispo, CA, United States

**Keywords:** ICDAS, intraoral camera, maternal transmission, oral health, toddler

## Abstract

**Objectives:**

Caries risk begins early in life, yet few studies use intraoral cameras to assess risk in toddlers or examine multiple contributing factors. This study aimed to address this gap and conduct intraoral camera-based assessment of caries and evaluate multiple toddler risk factors, including microbial profiles, oral hygiene practices, and potential maternal transmission.

**Methods:**

Cross-sectional data from 21 maternal–child dyads included intraoral camera assessments coded with an adapted International Caries Detection and Assessment System, saliva-based microbial tests for *Streptococcus mutans* (SM) and *Lactobacillus* (LB), and validated surveys of hygiene practices.

**Results:**

Among toddlers (*N* = 21; mean age 3.9 years), intraoral camera use was well tolerated and produced images adequate for assessment. Reliability was good for sound teeth [Intraclass correlation coefficient (ICC) = 0.80], moderate for initial caries (ICC = 0.72), and poor for moderate-to-severe caries (ICC = 0.13). Most teeth were sound (89.0%), with 9.8% mild and 1.1% moderate decay. SM was detected in 9/20 (45.0%) and LB in 4/21 (19.0%) of toddlers. While 81% brushed twice daily, few flossed (14.3%) or used mouthwash (19%); 42.1% consumed soda at least weekly and 19% ate fast food weekly. More frequent brushing was associated with lower SM (Cramer's *V* = 0.54; *p* = .02) and LB (*V* = 0.48; *p* = .043). Mothers (*N* = 21; mean age 32.1) had 38.0% of teeth coded as sound, 45.0% with initial caries, and 14.0% with moderate-to-severe decay; 9/21 (42.9%) tested positive for LB and the same proportion tested positive for SM. Most brushed twice daily (81%), with 47.6% flossing and 38.1% using mouthwash. Maternal and toddler brushing frequency (*V* = 0.68; *p* = .02), fast-food intake (*V* = 0.56; *p* = .01), and microbial positivity (SM: *V* = 0.93; *p* = .001; LB: *V* = 0.54; *p* = .02) were strongly related.

**Conclusions:**

Intraoral cameras with adapted ICDAS scoring were feasible for assessing caries risk in toddlers. Significant concordance between maternal and child microbial profiles suggests a potential maternal influence on early caries risk. Larger longitudinal studies are needed to clarify transmission pathways and inform prevention strategies.

## Introduction

Early childhood caries affects 28% of toddlers and is predictive of significant dental problems later in life (1, 2). Interventions to prevent early childhood caries have focused on promoting exposure to fluoride, via fluoridated water, consistent daily brushing with fluoride toothpaste, and application of fluoride varnish (3); and frequent visits to dental providers (4). Although fluoride toothpaste and varnish have been shown to be effective both systemically and topically to prevent caries in children, adherence to recommendations and access to care (1, 5) remain problematic. Application of antimicrobials such as silver diamine fluoride also appear highly effective in preventing caries in youth (6, 7), but this method causes damaged areas of the teeth to turn black (6). Researchers (8), medical providers (9, 10), and government agencies (11) have called for identification of novel targets for preventing caries, starting early in life.

In assessing caries risk in toddlers, most studies to date have relied on dental records. Newer technologies, such as intraoral cameras (12, 13), offer promise for easier and more standardized assessment, though their feasibility in toddlers remains untested. While the International Caries Detection and Assessment System (ICDAS) is well-validated in adults, it has been rarely used in children and has not been formally adapted for intraoral camera applications (12).

In toddlers, caries risk is multifactorial, involving biological, behavioral, and social determinants. Socioeconomic status is a known correlate, with children from lower-income households showing greater prevalence of caries, likely reflecting limited access to dental care (14). Lower frequency of dental visits is consistently associated with higher caries risk (15). Other commonly reported risk factors in toddlers include overgrowth of cariogenic microorganisms, including *Streptococcus mutans* (SM) and *Lactobacillus species* (LB) (16). Other risk factors include child excess body weight (17) and unhealthy dietary exposures (18). For example, one study found that children fed more sweets, soft drinks, and snack foods during their first year of life had a significantly higher risk of developing caries (19). Feeding practices, such as prolonged bottle-feeding, frequent nighttime feeding, or early introduction of sugar-containing foods and drinks, have also been linked to higher caries risk (20). Finally, toddlers may have limited ability to independently maintain oral hygiene, making them reliant on caregivers for regular brushing and supervision of habits like pacifier use or bottle sharing, which can indirectly influence microbial transmission (21).

Toddlers risk of caries may also be influenced by parental behaviors and health status. Maternal intake of fermentable carbohydrates and high rates of snacking (2, 22–24), for example, can increase maternal levels of SM and LB (2, 22). Parental elevated levels of SM and LB may translate into risk of caries in children via kissing, saliva transfer (e.g., sharing utensils) (21), oral contact, and premastication (25). Some studies suggested that improving maternal oral health during pregnancy may have protective effects on offspring oral health (26, 27). However, the available literature is limited and often conflicting (28–31). Additional research is needed to understand whether and how maternal oral health relates to child caries risk.

The primary goal of this study was to assess risk factors for caries in toddlers and to evaluate the feasibility of using intraoral cameras and an adapted ICDAS system for detecting and coding caries in this age group. A secondary aim was to explore how maternal characteristics, including tooth health and microbial profiles, might correlate with toddler caries risk. We hypothesized that intraoral cameras and adapted ICDAS coding would be feasible for use in toddlers, and that toddler caries risk factors would be positively correlated with maternal caries risk.

## Methods

Oral Health in Kids was a cross-sectional study. Recruitment occurred between August 2018 and June 2019 in Central Coast, California. A convenience sampling approach was used, with recruitment methods included via the research center's registry and flyers disseminated to participants in ongoing (non-intervention) studies and to local schools, pediatrician offices, and dental offices. This approach was selected to efficiently recruit mother–child dyads from the surrounding community for a feasibility-focused investigation of intraoral imaging and microbial sampling procedures.

Participant eligibility was based on self-report. To be eligible, mothers had to be ≥18 years, non-pregnant, and English or Spanish speaking. Children were required to be biological children of the participating mother and be 3–5 years of age. These criteria were selected to ensure inclusion of adult caregivers able to provide informed consent and to focus on preschool-aged children, a developmental period characterized by early primary dentition and increased susceptibility to early childhood caries. Participants were ineligible if they had any recent surgeries or took antibiotics in the two weeks before their scheduled visit. Antibiotic use and recent surgical procedures were exclusion criteria because they could alter oral microbial profiles and potentially bias assessment of SM and LB colonization.

Procedures were approved by the California Polytechnic State University Institutional Review Board (2018-137-CP; 4/3/2018–1/22/2022), and all participants provided written informed consent. The study followed the ethical standards of the California Polytechnic State University San Luis Obispo.

The single assessment visit was conducted with mothers and toddlers by trained research assistants. During the visit, mothers completed a questionnaire, and anthropometrics, saliva samples, and intraoral video recordings were obtained for both mothers and toddlers. At the end of the visit, all participants received written and verbal information about recommendations for healthy oral habits in adults and children based on the American Dental Association's recommendations (32). Participants also received a toothbrush and stickers and $30 for completing the assessment.

To assess tooth health, children's and mother's teeth were video recorded by trained research assistants using an intraoral camera (Carestream CS1500, Carestream Dental, Atlanta, GA, USA), which captures high-resolution images with integrated LED illumination. Video recordings captured three surfaces per tooth (buccal, occlusal/incisal, and lingual). Two trained research assistants collected these measures following standard intraoral photography protocols to capture the buccal, occlusal/incisal and lingual surfaces of each tooth starting with teeth 1–16 (top) and then 17–32 (bottom) (33). For quality control, the research assistants spent approximately 1 s capturing each tooth and held the camera 1 cm away from the tooth surface. If the camera fogged up, the lens was wiped with gauze, and participants were asked to breathe through their nose. When capturing the buccal (cheek) surface, the toddler and mother were asked to open their mouth only slightly. As needed for toddlers, staff provided sticker rewards, a parent-approved movie, or had the mothers hold the toddler on their laps while recording the child's teeth. Intraoral imaging was typically completed within approximately 2–4 min per participant.

The ICDAS was used to assess the severity of caries (Supplementary Table 1) (34). ICDAS is an internationally validated and publicly available caries detection system that does not require licensing for research use. The data was grouped into three overview classes: Sound tooth surfaces (ICDAS Score 0) Initial caries (ICDAS Score 1 or 2), moderate to severe caries (ICDAS Score 3 to 6). Two different raters coded the toddlers’ and mothers’ teeth. The coders met prior to assigning scores and established which teeth were present in the participant's mouth. Once tooth presence was established, the coders scored independently and without collaboration. Interrater reliability was assessed using intraclass correlation coefficients (ICC), and these were interpreted using thresholds: <0.50 = poor, 0.50–0.75 = moderate, 0.75–0.90 = good, and >0.90 = excellent reliability.

For the assessment of oral microbial profile, participants were asked to refrain from eating or drinking beverages other than water in the hour leading up to the visit. In children*,* 4–5 mL of saliva was collected by the research assistant or the toddler's mother. For toddlers, saliva collection was gamified into a competition between who could spit more between the mother and toddler. If the child was 4 years or older, research assistants asked the mother if the toddler chewed gum regularly. If so and with parent permission, the research assistants offered the child paraffin wax to simulate saliva. If not, to stimulate the secretion of saliva, children's teeth were brushed without toothpaste, and the child was asked to spit into a polypropylene tube. If this did not yield saliva, a sterile cotton gauze strip or dental roll was massaged in the buccal and lingual areas of the mouth until saturated and transferred immediately to a syringe. Saliva was expressed into a conical propylene tube until approximately 5 mL was obtained and then transferred to the research center's laboratory for microbiological analyses. In mothers, a sample of 4–5 mL stimulated whole saliva was collected and SM and LB were measured. Specifically, each mother was asked to chew a paraffin pellet to stimulate the secretion of saliva and to transfer bacteria from tooth surfaces into the saliva. Participants then spit the saliva into collection tubes for processing and analysis. All samples were subjected to microbiological analyses within 4 h of collection.

Saliva samples were serially diluted in sterile saline (10^−1^–10^−5^) and 0.1 mL aliquots were plated on Lactobacillus selective agar and Tryptone Yeast Extract Cystine Sucrose Bacitracin (TYCSB) agar. Plates were incubated for 48 h at 37 °C under reduced atmospheric oxygen conditions. Resulting colony-forming units (CFUs) on plates containing 30–300 CFU were enumerated, and CFU/mL was calculated using a standard formula. Growth on TYCSB followed by Gram staining was used to confirm the identity of SM colonies in saliva samples. Colonies on Lactobacillus selective agar were identified as LB through Gram staining and Biolog Gen III microplate phenotyping. Using simulated and autoclave sterilized saliva samples inoculated with *Lactobacillus* and *S. mutans* cells, we estimated that the limit of detection for each target was 10 CFU/mL.

Maternal and child brushing and flossing habits, toothpaste use, prescription fluoride drops, prescription fluoride tablets, and frequency of visits to dentist and dental procedures were measured based on the National Health and Nutrition Examination (NHANES) oral health survey (35). NHANES survey instruments are publicly available and were used in accordance with CDC guidelines. Mothers completed a validated Eating Behaviors Questionnaire (36) to report frequency of child consumption of sugar sweetened beverages and fast food. Scoring procedures followed the published methodology for this instrument. Breastfeeding and timing of introduction of complementary feeding was measured using three questions adapted from the Southampton Women's Survey and CDC Infant Feeding Practices study (37). To determine frequency of premastication, a measure from the Centers for Disease Control and Prevention, Infant Feeding and Breast Feeding questionnaire (1) was used, and utensil and cup sharing was measured using a single question.

Demographic assessments included mother and child age, family income, race/ethnicity, education, marital status, and number of people living in the home. Weight was assessed in lightweight clothing using a calibrated scale. Height was measured without shoes. Maternal and child height and weight was used to calculate BMI in mothers and BMI centiles in children using CDC Growth Charts (38).

### Statistics

Descriptive statistics, including means, standard deviations, and percentages, were used to examine participant demographics and caries risk factors. As this was a feasibility study designed to assess procedural acceptability and generate preliminary estimates, a formal *a priori* sample size calculation was not performed; the final sample size was determined by recruitment feasibility during the study period. Inter-rater reliability for ICDAS coding was assessed using the ICC. Paired samples correlations and t-tests were used to examine associations between children and maternal dyads on continuous variables and chi-square test with Cramér's *V* was used to examine associations between children and maternal dyads on categorical variables. Among toddlers, ANOVAs tested differences in caries, LB, and SM across categories of oral hygiene and dietary practices (e.g., tooth brushing frequency, mouthwash use, soda and fast-food consumption) as well as zBMI category. Chi-square tests with Cramer's *V* were used to examine associations between these categorical behaviors and dichotomous outcomes for LB, SM, and tooth health. Data were analyzed using IBM SPSS Statistics, version 29.0.2 (2023).

## Results

**Figure 1:**
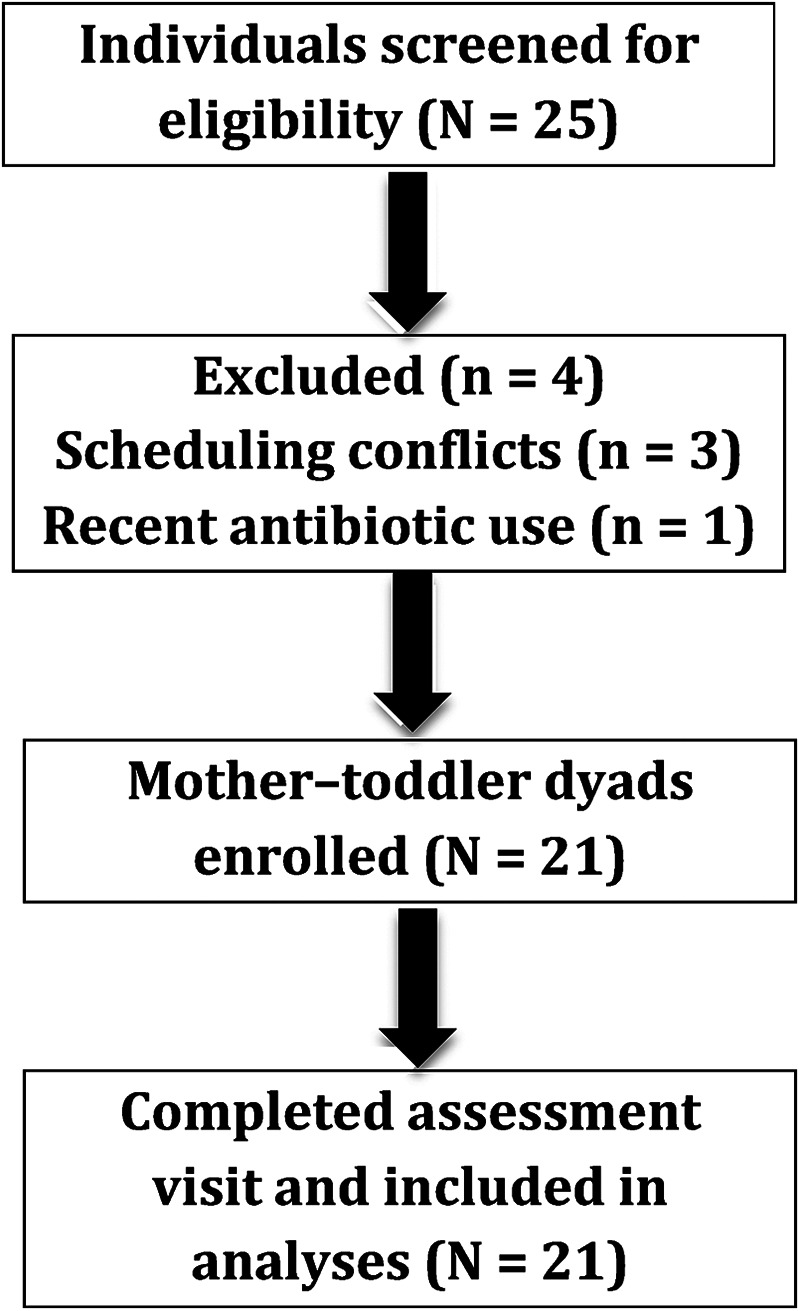
Recruitment flow. Flow diagram illustrates participant recruitment and enrollment. Of 25 individuals screened for eligibiblity, 4 were excluded (3 due to scheduling conflicts and due to recent antibiotic use). A total of 21 mother-toddler dyads were enrolled and completed assessment visit and were included in the final analyses. The figure was created using Microsoft PowerPonit (Microsoft 365, Version 16.106.2).

**Figure 2:**
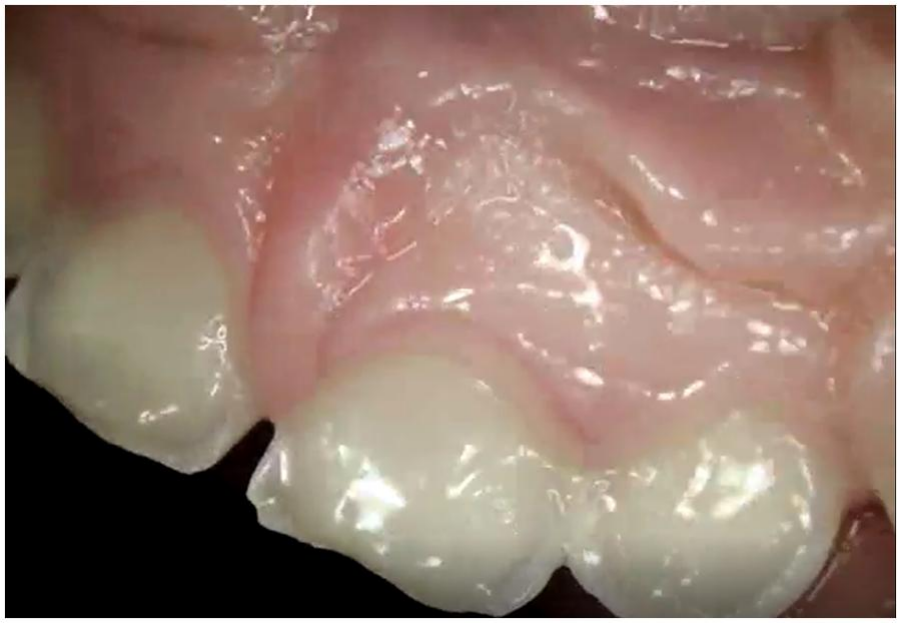
Lingual view of the maxillary central incisors of the mother's toddler.

Of the 25 people screened for this study, 21 mother/toddler dyads met criteria and were enrolled (Figure 1). Main reasons for ineligibility included scheduling conflicts (*n* = 3), and antibiotic use (*n* = 1). Toddlers had a mean age of 3.9 years; 40% had obesity, and 71.4% were identified by their mothers as Hispanic (Table 1). Videos were obtained and deemed interpretable for all toddlers (*N* = 21). Representative intraoral photographs from a toddler is shown in Figure 2. Inter-rater reliability for toddlers teeth coding was good for teeth coded as sound (ICC = 0.80; *p* < .001) and moderate for initial caries (ICC = 0.72; *p* = .003), but poor for toddlers with moderate-to-severe caries (ICC = 0.13; *p* = .60), likely reflecting a single rater disagreement and the low prevalence (1.1%) of moderate-to-severe lesions in the teeth of toddlers in this sample.

**Table 1:** Participant demographics.

Characteristic	Toddlers *N* = 21	Mothers *N* = 21
Age, mean (SD) years	3.9 (0.9)	32.1 (5.4)
Ethnicity, % Hispanic (no./no.)	15/21 (71.4%)	13/21 (61.9%)
Income < $40,000, % (no./no.)	N/A	7/20 (35%)
Education ≥ college, % (no./no.)	N/A	9/21 (42.9%)
Number of family members in household, mean (SD)	3.9 (1.0)	3.9 (1.0)
Average annual household family income range ($) (*N* = 20)	N/A	50–59.999
Weight, kg, mean (SD)	16.8 (2.5)	81.2 (22.0) (*n* = 20)
zBMI, mean (SD) (*n* = 20)	0.5 (0.8)	
BMI, mean (SD)		31.1 (7.8)
BMI classification		
Normal BMI, % (no./no.)	12/20 (60.0%)	3/21 (14.3%)
Overweight/obesity, % (no./no.)	8/20 (40.0%)	18/21 (85.7%)
Gestational weight gain (kg) (SD)	N/A	11.5 (6.7)
Took antibiotics within last 30 days prior to saliva collection, No. (%)	2/20 (9.5%)	1/20 (4.8%)

Values are presented as mean (SD) or n/N (%). zBMI = age- and sex-specific body mass index z-score calculated using CDC growth charts. BMI, body mass index (kg/m²). N reflects the number of participants with available data for each variable.

On average, toddlers had 19.8 teeth (SD = 0.51), with 89.0% of teeth [17.6 (1.7)] coded as sound (Table 2). Smaller proportions of teeth were coded as having mild decay [9.8%; 2.0 (1.7)] or moderate-to-severe decay [1.1%; 0.2 (0.6)]. Examining microbial results, 45% (9/20) of toddlers tested positive for SM, and 19% (4/21) tested positive for LB, with average concentrations of 43,800 CFU/mL (SD = 95,630) and 1,320 CFU/mL (SD = 3,390), respectively. The proportion of teeth exhibiting initial caries lesions (ICDAS 1–2) was not significantly correlated with LB or SM CFU/mL concentrations. Per maternal reports, most toddlers (81%) brushed their teeth twice or more per day; 14.3% flossed at least once per day and 19% rinsed with mouthwash once per day. Overall, 42.1% of toddlers consumed soda at least weekly, and 19.0% consumed fast-food on at least a weekly basis. More frequent brushing was related to a lower frequency of positive SM (*V* = 0.54; *p* = .02) and lower LB (*V* = 0.48; *p* = .043). No meaningful associations were observed between toddler brushing or mouthwash use and zBMI, soft drink or fast-food consumption, nor between these behaviors and LB, SM, or tooth health categories.

**Table 2:** Tooth decay and microbial profiles in toddlers and their mothers.

Variable	Toddler (*N* = 21)	Mother (*N* = 21)	Paired samples Pearson correlations (r) and paired t-tests
Teeth with mild, moderate, or no tooth decay			
Number of moderate-severe caries teeth (ICDAS = 3–6), mean (SD)	0.2 (0.58)	3.8 (4.3)	*r* = 0.15; *p* = 0.26
Number of initial caries teeth (ICDAS = 1–2), mean (SD)	2.0 (1.7)	12.4 (6.3)	*r* = −0.36; *p* = 0.06
Number of sound teeth (ICDAS = 0), mean (SD)	17.6 (1.7)	10.7 (5.5)	*r* = −0.17; *p* = 0.22
Average proportion (%) of teeth with ICDAS > 3			
Moderate to severe range (scores = 3–6), mean (SD)	1.1 (3.0)	14.0 (16.0)	*r* = 0.14; *p* = 0.55
Average proportion (%) of teeth with ICDAS score range of 1–2 (Mild “initial caries” range), mean (SD)	9.8 (8.3)	45.0 (23.0)	*r* = −0.34; *p* = 0.13
Average proportion (%) of teeth with ICDAS score of 0 (sound), mean % (SD)	89.0 (8.9)	38.0 (20.0)	*r* = −.021; *p* = 0.34
Oral microbial profiles			
CFU/mL for SM colonies on TYCSB media, mean (SD)	43,800 (95,630)	16,500 (9,300)	*r* = −0.19; *p* = 0.71
CFU/mL for LB colonies on selective agar media, mean (SD)	1,320 (3,390)	30,240 (63,010)	*r* = 0.44; *p* = 0.06
Participants with a positive SM result; no./no (%)	9/20 (45.0%)	9/21 (42.9%)	***V*** **=** **0.93; *p*** **=** **0.001**^a^
Participants with a positive LB result; no./no. (%)	4/21 (19.0%)	9/21 (42.9%)	***V*** **=** **0.54; *p*** **=** **0.02**^a^

ICDAS, International Caries Detection and Assessment System; CFU/mL, colony forming units per milliliter; SM, Streptococcus mutans; LB, Lactobacillus; TYCSB, Tryptone Yeast Extract Cystine Sucrose Bacitracin medium.

Values are presented as mean (SD) or *n*/*N* (%). Pearson correlation coefficients (*r*) were used for continuous mother–toddler comparisons; paired *t*-tests were conducted where appropriate. Categorical comparisons were evaluated using chi-square tests with Cramér's *V* effect sizes.

Bold indicates *p* < 0.05.

a Indicates results from chi-square test with Cramér's *V*.

**Figure 3:**
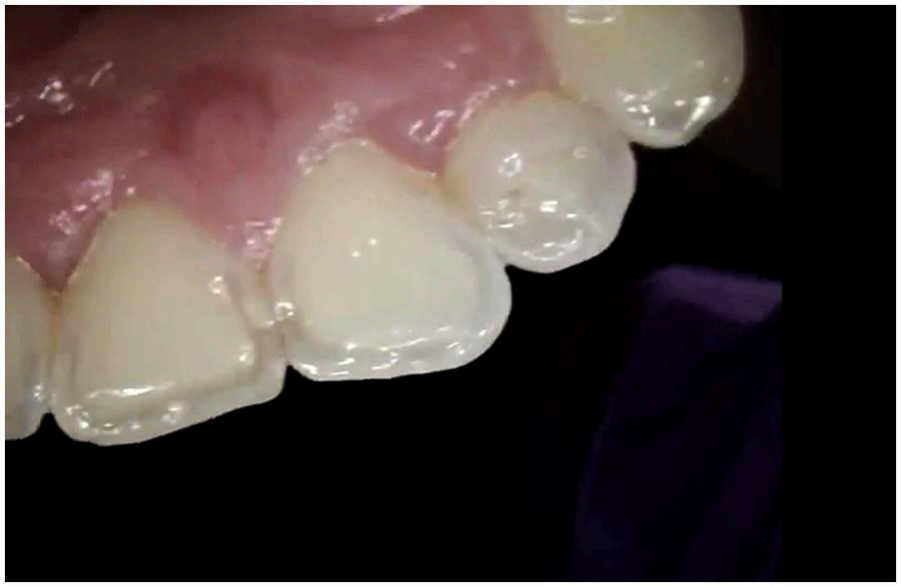
Lingual view of the maxillary central incisors of a mother participant.

Mothers had a mean age of 32.1 years; 85.7% were classified as having overweight/obesity, and 61.9% of mothers identified as Hispanic. Videos were obtained and deemed interpretable for all mothers (*N* = 21). Representative intraoral photographs from a mother is shown in Figure 3. Inter-rater reliability was moderate for mothers’ teeth coded as sound (ICC = 0.69; *p* = .006), initial caries (ICC = 0.81; *p* < .001), and moderate-to-severe caries (ICC = 0.92; *p* < .001). About 38.0% [10.7 (5.5)] of maternal teeth were coded as sound; most were coded as having mild caries (45.1%; *M* = 12.4, SD = 6.3); and 14.3% (*M* = 3.8, SD = 4.3) had moderate-to-severe decay. In full, 9/21 (42.9%) of mothers tested positive for LB and 9/21 (42.9%) tested positive for SM, with average concentrations of LB being 30,240 CFU/mL (SD = 63,010) and SM levels being 16,500 CFU/mL (SD = 9,300). The proportion of teeth exhibiting initial caries lesions (ICDAS 1–2) was positively correlated with SM concentrations (CFU/mL) (*r* = 0.577, *p* = 0.006). In contrast, no significant association was observed between the proportion of teeth with initial caries lesions and LB concentrations (*p* = 0.67). Mothers reported brushing twice or more per day (81%); 7.6% reported flossing daily, and 38.1% reported daily use of mouthwash. A significant proportion reported weekly or more frequent fast-food consumption (42.9%) and weekly or more frequent soda intake (47.6%) (Table 3).

**Table 3:** Oral hygiene and dietary risk factors for caries in toddlers and mothers (n/n, %).

Variable	Toddlers *N* = 21	Mothers *N* = 21	Cramér's *V* & chi-Square
Oral Hygiene			
Tooth Brushing			***V* = 0.68; *p* = 0.02**
Brush teeth less than once per day, No./No. (%)	0/21 (0%)	1/21 (4.8%)	
Brush teeth once per day, No./No. (%)	4/21 (19%)	3/21 (14.3%)	
Brush teeth twice or more per day, No./No. (%)	17/21 (81%)	17/21 (81%)	
Flossing			*V* = 0.55; *p* = 0.06
Never floss teeth, No./No. (%)	8/21 (38.1%)	1/21 (4.8%)	
Floss teeth less than once per day, No./No. (%)	10/21 (47.6%)	10/21 (47.6%)	
Floss teeth at least once per day, No./No. (%)	3/21 (14.3%)	10/21 (47.6%)	
Mouthwash			
Never rinse with mouthwash, No./No. (%)	16/21 (76.2%)	5/21 (23.8%)	*V* = 0.35; *p* = 0.52
Rinse with mouthwash less than once per day, No./No. (%)	1/21 (4.8%)	8/21 (38.1%)	
Rinse with mouthwash once or more per day, No./No. (%)	4/21 (19.0%)	8/21 (38.1%)	
Fluoride			
Never use fluoride drops or tablets, No./No. (%)	19/19 (100%)	21/21 (100%)	N/A
Dietary factors			
Drink soda or pop 1 or more per week, No./No. (%)	8/19 (42.1%)	10/21 (47.6%)	*V* = 0.26; *p* = 0.26
Consume fast food 1 or more per week, No./No. (%)	4/21 (19.0%)	9/21 (42.9%)	***V* = 0.56; *p* = 0.01**
Currently drink alcohol, No./No. (%)	N/A	10/21 (47.6%)	N/A
Premastication No./No. (%)	N/A	1/21 (4.8%)	N/A
Child breastfed during infancy, No./No. (%)	18/21 (85.7%)	N/A	N/A
Share utensils and drinking cups with child, No./No. (%)	N/A	19/21 (90.4%)	N/A
Currently smoke tobacco, No./No. (%)	N/A	2/21 (9.5%)	N/A

No., number of participants; N/A, not applicable; statistical comparison not performed; *V*, Cramér's *V*.

Values are presented as *n*/*N* (%). Associations between toddlers and mothers were evaluated using chi-square tests with Cramér's *V* effect sizes.

Bold indicates *p* < 0.05.

Proportions of toddlers and mothers with positive SM and LB were highly related (*V* = 0.93; *p* = 0.001; *V* = 0.54; *p* = 0.02, respectively; Table 2). Correlations within dyads were also observed for tooth brushing frequency (*V* = 0.68; *p* = 0.02) and fast-food consumption (*V* = 0.56; *p* = 0.01; Table 3). Correlations between maternal and child tooth health, including the number of sound teeth, initial caries, and moderate to severe caries, were not significant (Table 2).

## Discussion

This study demonstrated the feasibility of assessing caries risk in toddlers using intraoral cameras with an adapted ICDAS system, in combination with microbial sampling and surveys of oral hygiene practices. Intraoral imaging yielded reliable scoring for sound and initial caries, and most toddlers were coded as having sound teeth with only a small proportion showing early decay. Several caries risk factors were prevalent in the toddlers in this study, including overweight/obesity, positive tests for SM and LB, and frequent soda consumption, and protective behaviors included prevalent twice-daily tooth brushing. Notably, brushing frequency was associated with a lower likelihood of SM and LB detection, underscoring the potential impact of early oral hygiene on lower microbial risk factors for caries in toddlers.

The use of intraoral cameras in young children can be challenging because of fear of a foreign object in the mouth and difficulty keeping the mouth open. One study reported that children preferred their mouths to be photographed with smartphones, although intraoral cameras required less time (39). Another study found that intraoral cameras were feasible for telehealth dentistry, with children more accepting when the camera was used by their parents (40). In our study, an intraoral camera produced clear videos that were interpretable for ICDAS scoring.

ICDAS is a standardized method for measuring dental caries that has demonstrated accuracy and reproducibility (41). Previous studies have compared intraoral camera images against ICDAS scoring, but to our knowledge, none have used ICDAS to score teeth from videos captured with intraoral cameras and in toddlers (12, 42). Adapting ICDAS for intraoral camera use allowed us to code the teeth reliably in toddlers. Inter-rater reliability was acceptable for toddlers’ teeth coded as sound and for initial caries but poor for toddlers’ moderate-to-severe caries, likely reflecting the low prevalence of these lesions in the sample and a single rater disagreement. Future research with larger sample sizes is needed and should support coder training to improve identification of moderate to severe caries in toddlers. Future research is also needed to validate ICDAS scoring from intraoral camera videos against provider-performed clinical examinations in this age group.

This study found that most toddlers brushed twice per day, and brushing frequency was associated with a lower occurrence of oral SM and LB bacteria. The benefits of regular toothbrushing in toddlers is well established (22), and reducing SM and LB could improve oral health in children. Of course, caries risk prediction is strongest when microbial indicators are considered together with behavioral and dietary factors (22, 43).

In the current study, nearly half of toddlers consumed soda at least weekly, and one in five had weekly or more frequent fast food, but these behaviors were not significantly associated with SM or LB status, which could reflect limited sample size. Taken together, findings from the current study reinforce the importance of daily brushing in reducing bacterial load in toddlers. Type of toothpaste (fluoridated and non-fluoridated) should be included in future studies. Future research in larger sample sizes could examine lingual brushing, as the tongue can be an important source of cariogenic microbes, and strategies to pair brushing with flossing, which were less common in this sample, and promote early oral hygiene behaviors to reduce microbial risk of future caries.

**Figure 4:**
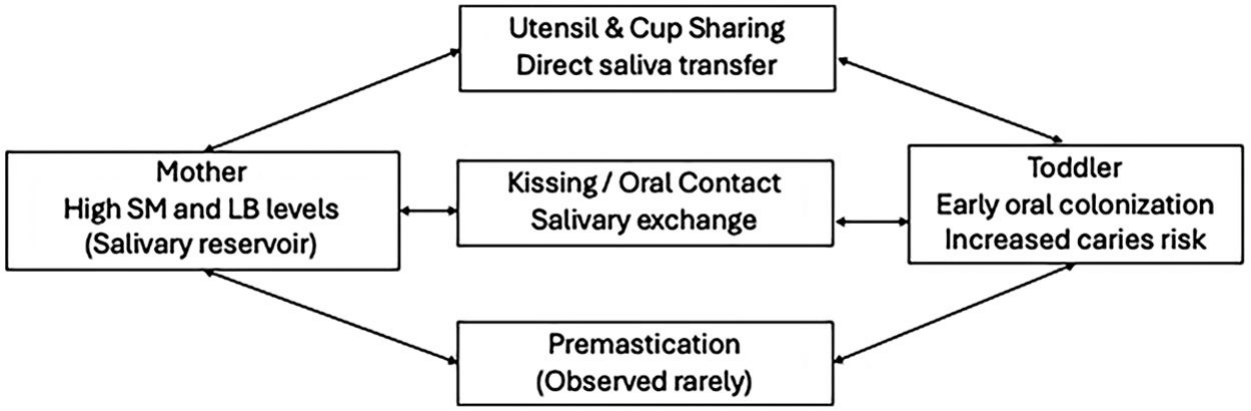
Potential mechanisms of Maternal-to-Child bacterial transmission. The figure illustrates hypothesized mechanisms of vertical transmission of cariogenic bacteria from mother to toddler. Elevated maternal *Streptococcus mutans* (SM) and Lactobacillus (LB) levels may serve as a salivary reservoir, with transmission occuring via utensils and cup sharing, kisssing/oral contact, and premastication, leading to early microbial colonization and increased caries risk in the child. The figure was created using Microsoft PowerPoint (Microsoft 365, Version 16.106.2).

A secondary aim of the study was to explore how maternal characteristics, including oral health status and microbial profiles, correlated with toddler caries risk. Findings indicated that mother–toddler dyads had significantly correlated SM and LB statuses, suggesting a co-shared microbial environment. Additional research is needed to determine whether and how maternal SM and LB microorganisms in mother may shape the child's microbial environment. These findings are consistent with the well-documented phenomenon of “vertical transmission,” in which cariogenic bacteria—particularly *Streptococcus mutans*—are transmitted from mother to child through salivary contact (Figure 4). High maternal salivary levels of SM have been shown in prior studies to predict earlier colonization and higher caries risk in offspring. In our cohort, the strong concordance of SM and LB between mothers and toddlers suggests that elevated maternal bacterial burden may have directly translated into increased microbial risk in children.

Among mothers, the proportion of teeth with initial caries lesions was positively correlated with salivary SM concentrations. In contrast, no significant association was observed between maternal initial caries lesions and LB concentrations. Higher SM burden may be associated with greater prevalence of early-stage caries in adults and support the biological plausibility that mothers with higher cariogenic bacterial loads may contribute to the shared microbial patterns observed in mother–child dyads. However, the relatively small sample size and low prevalence of moderate-to-severe caries in both mothers and toddlers limited these analyses.

The vast majority (>90%) of mothers in this study reported sharing utensils and drinking cups with their child, which may help explain the correlations observed in dyads for SM and LB. Such frequent saliva-sharing behaviors provide a biologically plausible pathway for bacterial transmission, as shared utensil use has been identified as a key mechanism facilitating early colonization of cariogenic organisms. Given the extremely high prevalence of utensil and cup sharing in our sample, vertical transmission through routine daily interactions is a likely contributor to the microbial similarities observed between mothers and toddlers. “Vertical transmission,” wherein cavity-causing bacteria from affected mothers are transmitted to their children, has been documented to occur via kissing, saliva transfer (e.g., sharing utensils), oral contact, and premastication (21, 44). A nonrandom sample of 68 caregivers in Nebraska reported a 65% prevalence of premastication (45), while data from the 2005–2007 Infant Feeding Practices Study II of approximately 1,600 mothers suggested a much lower but still notable prevalence of about 14% (46). These comparisons refer to different historical periods (∼1998 vs. 2007) and different populations, with potentially different cultural contexts, which could limit the comparability of premastication prevalences. In the current study, premastication was reported by only one mother.

Importantly, while premastication was rare in our cohort, the near-universal practice of sharing utensils and cups suggests that everyday feeding behaviors may represent a more common and sustained route of bacterial transmission than premastication alone. This reinforces the need to consider maternal microbial load and household saliva-sharing behaviors together when assessing toddler caries risk. Taken together, these findings, along with the observed biological correlations, provide additional support for potential for co-transmission of SM and LB within mother–toddler dyads. Improving maternal oral health starting during pregnancy may have protective effects on offspring oral health through 24 months (26, 27), potentially by reducing maternal reservoirs of cariogenic bacteria available for vertical transmission, though whether maternal oral health interventions have positive effects on toddlers after 24 months requires further study.

Mothers and children shared some behavioral patterns, such as toothbrushing frequency and fast-food consumption, suggesting a potential maternal influence on child behaviors related to caries risk as well. Research in 674 mother–child dyads in Brazil found significant correlations between maternal and child sugar consumption (SC = 0.236) (47). Future research should consider assessing other caregivers and using more extensive and validated instruments of maternal and child food intake.

The current study did not find an association between prior breastfeeding and toddler caries risk. Whether breastfeeding or formula feeding affects caries risk remains debated. Some evidence suggests that breastfeeding, which is less common among women with obesity, provides immunoglobulin G with antibacterial activity against SM (48). In contrast, “nursing caries” have been reported when children are put to sleep while breastfeeding, with residual milk in the mouth increasing caries risk (49). Similar risks have been observed in children put to bed with bottles containing formula, milk, juice, or other sugary drinks (49).

To our knowledge, this is the first study to test the feasibility of using intraoral cameras with ICDAS scoring in toddlers. The study included a diverse sample with several caries risk factors, including obesity experienced in 40% of toddlers and more than 85% of mothers; however, findings may not be generalizable to other populations. The use of a convenience sample and the relatively small sample size limit statistical power and may increase the risk of Type II error. As a feasibility study, the sample size was small, and the wide variability in CFUs for SM and LB suggests that individual differences may have disproportionately influenced group means. Additionally, the cross-sectional design precludes determination of temporal or causal relationships between maternal microbial burden, behavioral factors, and toddler caries risk. Behavioral measures were based on maternal self-report and may be subject to recall or social desirability bias. Microbial sampling was conducted at a single time point, which may not fully capture dynamic fluctuations in oral bacterial colonization. Future studies should standardize moisture conditions and directly compare dry and wet imaging against clinical examinations. Future studies should also evaluate other caregivers, including fathers, to inform family-based interventions for caries prevention management in early childhood. Also, plates were incubated for 48 h, per manufacturer's recommendations, but could be monitored longer to detect slower growing strains. An expanded study could include lingual biofilm sampling to better understand how this important source of cariogenic microbes might be influenced by the variables we measured. Furthermore, as the cost of DNA sequencing analysis becomes more affordable, we could greatly expand the breadth of microbiological analysis by studying temporal variation in metagenomes. Also, longitudinal studies are needed to clarify the directionality of microbial transmission within mother–toddler dyads and to determine whether reductions in maternal cariogenic bacterial load translate into measurable reductions in early childhood caries. Future studies should also evaluate the validity of intraoral camera–based ICDAS scoring against clinical dental examinations in toddlers and explore intervention strategies targeting both maternal oral health and early childhood hygiene behaviors.

## Conclusion

This feasibility study supports the use of intraoral cameras with an adapted ICDAS protocol to assess early caries risk in toddlers. Although most toddlers had sound dentition, multiple behavioral and microbial risk factors were present, and concordance of Streptococcus mutans and Lactobacillus between mothers and children suggests a shared microbial environment. Twice-daily toothbrushing was associated with lower detection of cariogenic bacteria, underscoring the importance of early oral hygiene. However, findings are limited by the small sample, cross-sectional design, and reliance on self-reported behaviors. Larger, longitudinal studies are needed to confirm these findings and clarify maternal–child influences on early caries risk.

## Data Availability

The raw data supporting the conclusions of this article will be made available by the authors, without undue reservation.
