# Supplementary material for: Caries risk in toddlers: assessing oral health, microbial factors, and maternal transmission
Source: Front Dent Med. 2026 Jun 22;7:1847228. doi: 10.3389/fdmed.2026.1847228 (PMC13333731; doi:10.3389/fdmed.2026.1847228)
Supplement: Supplementary file 1 [file Table1.docx]

| **Supplementary Table 1. Two-Number International Caries Detection and Assessment System (ICDAS) Method for Coding Caries Risk** | |
| --- | --- |
| **CODE** | **DESCRIPTION** |
| Tooth Status - First Number - | |
| 0 | Sound (use with the codes for primary caries) |
| 1 | Sealant, partial |
| 2 | Sealant, full |
| 3 | Tooth colored restoration |
| 4 | Amalgam restoration |
| 5 | Stainless steel crown |
| 6 | Porcelain, gold, PFM crown or veneer |
| 7 | Lost or broken restoration |
| 8 | Temporary restoration |
| 9 | Used for the following conditions |
| 97 | Tooth extracted because of caries (all tooth surfaces will be coded 97) |
| 98 | Tooth extracted for reasons other than caries (all tooth surfaces coded 98) |
| 99 | Uncodable (all tooth surfaces coded 99) |
| Tooth Status - Second Number | |
| 0 | Tooth surface appears normal, with no signs of decay. Enamel looks healthy in color, shine, and translucency. If restorations or sealants are present, they show no evidence of caries. |
| 1 | Very early enamel change is visible as a white spot after drying the tooth for several seconds. The change is limited to small pit or fissure areas and not due to other surface variations. |
| 2 | Clear change in enamel is visible even without drying. White or brown discoloration extends beyond the natural fissure or groove pattern. |
| 3 | Initial breakdown of enamel is seen, but dentin is not yet exposed or shadowed underneath. Chalky or brown discoloration may appear wider than expected in the fissure or pit, with visible surface loss confined to enamel. |
| 4 | Dark shadow from underlying dentin is visible beneath an otherwise intact enamel surface. The enamel may or may not show localized breakdown. The shadow often appears grey, blue, or brown. |
| 5 | A distinct cavity has formed with dentin exposed. Enamel loss reveals darker tissue beneath, and cavitation is visible at pits or fissures. |
| 6 | A large cavity is present, involving at least half of the tooth surface and often extending toward the pulp. The cavity is deep and wide, with dentin clearly visible on the walls and base. |
| ICDAS Coding reference is from Ismail et al. | |
